# The anti-inflammatory effects of the tellurium redox modulating compound, AS101, are associated with regulation of NFκB signaling pathway and nitric oxide induction in macrophages

**DOI:** 10.1186/1476-9255-7-3

**Published:** 2010-01-20

**Authors:** Miri Brodsky, Gilad Halpert, Michael Albeck, Benjamin Sredni

**Affiliations:** 1 C.A.I.R. Institute, The Safdiè AIDS and Immunology Research Center, The Mina & Everard Goodman Faculty of Life Sciences, Bar-Ilan University, Ramat-Gan 52900, Israel; 2 Department of Chemistry, Faculty of Exact Sciences, Bar-Ilan University, Ramat-Gan 52900, Israel

## Abstract

**Background:**

LPS-activated macrophages produce mediators which are involved in inflammation and tissue injury, and especially those associated with endotoxic shock. The non toxic tellurium compound ammonium tri-chloro(dioxoethylene-O,O'-)tellurate, AS101, has been recently shown to exert profound anti-inflammatory properties in animal models, associated with its Te(IV) redox chemistry. This study explores the anti-inflammatory properties of AS101 with respect to modulation of inflammatory cytokines production and regulation of iNOS transcription and expression in activated macrophages via targeting the NFkB complex.

**Results:**

AS101 decreased production of IL-6 and in parallel down-regulated LPS-induced iNOS expression and NO secretion by macrophages. AS101 reduced IkB phosphorylation and degradation, and reduced NFkB nuclear translocalization, albeit these effects were exerted at different kinetics. Chromatin immunoprecipitation assays showed that AS101 treatment attenuated p50-subunit ability to bind DNA at the NFkB consensus site in the iNOS promotor following LPS induction.

**Conclusions:**

Besides AS101, the investigation of therapeutic activities of other tellurium(IV) compounds is scarce in the literature, although tellurium is the fourth most abundant trace element in the human body. Since IKK and NFkB may be regulated by thiol modifications, we may thus envisage, inview of our integrated results, that Te(IV) compounds, may have important roles in thiol redox biological activity in the human body and represent a new class of anti-inflammatory compounds.

## Introduction

Inflammation is the central feature of many pathophysiological conditions that occur in response to tissue injury and as part of host defenses against microorganisms. Macrophages are the main proinflammatory cells responsible for invading pathogens by releasing proinflammatory mediators such as IL-6, including the short lived free radical, NO[1]. During inflammatory processes, large amounts of NO generated by the inducible isoforms of NOS has been suggested to have beneficial microbicidal, antiviral and antitumoral effects; however, aberrant iNOS induction is involved in the pathophysiology of many human diseases[2]. Additionally, LPS-induced IL-6 production acts as an endogenous pyrogen in addition to its multiple effects on the immune system[3]. NFκB is one of the most ubiquitous transcription factors and functions as a central player in the chronic inflammatory diseases development, partly through IL-6[4,5] and iNOS expression [6-8]. Thus, discovery of inhibitors that preferentially target the binding of NFκB to its consensus DNA sequence would have important clinical applications. Moreover, NFκB activation is tightly linked with redox regulation since the DNA binding activity of oxidized NFκB is significantly diminished[9]. NFκB is present in the cytoplasm as an active heterotrimer consisting of p50, p65 and IkBα subunits. Upon activation of the complex, phosphorylation and degradation of IkBα exposes nuclear localization signals on the p50/p65 complex, leading to nuclear translocation and binding to specific regulated sequences in the DNA, thus controlling gene transcription[10].

AS101, a small non toxic organotellurium-IV compound, is a potent immunomodulator (*in-vitro *and *in-vivo*) with a variety of potential therapeutic applications [11-13]; it is currently being evaluated in PhaseII clinical trials in cancer patients. Accumulated evidence suggests that much of the biological activity of organotellurium compounds is directly related to their specific chemical interactions with endogenous thiols and may be important for manifestation of the biological function itself. Previously, we clarified several mechanistic aspects of this chemistry, and discussed its relationship to the biological activity of AS101[14]. If the reacting thiol is a cysteine residue, the reaction product may alter the biological activity of the target protein. The Te(IV)-thiol chemical bond may lead to conformational change or disulfide bond formation, possibly resulting in a loss of the biological activity, if the thiol residue is essential for that function. Indeed, we demonstrated that AS101 and other TeIV-compounds specifically inactivate cysteine proteases [14-16], while exhibiting no effect on the other families of serine-, aspartic- and metalloproteases, in good agreement with the predictions of their unique Te(IV)-thiol chemistry. Furthermore, the proteolytic activity of the inactivated cysteine proteases could be restored by reducing agents such as NaBH_4_, further supporting the suggestion that the inactivation process involves oxidation of the catalytic thiol to a disulfide[14]. Because of the Te(IV) valence of AS101, it can serve as a reducing or oxidizing agent, depending on the environmental oxidation milieu[17]. Previously we demonstrated that AS101 exerts anti-inflammatory effects in different *in-vivo *models through possible redox-mechanism with thiols[15,16,18]. In light of the thiol sensitive regulation of the NFkB pathway, this study explores if the redox traits of AS101 will enable its anti-inflammatory effects with respect to its ability to reduce pro-inflammatory cytokines and inhibit iNOS expression and NO release in LPS-stimulated RAW264.7 macrophages by targeting the NFκB activation pathway.

## Materials And Methods

### Cell Culture and Sample Treatment

The RAW264.7 murine macrophage cell line was grown at 37°C in DMEM medium supplemented with 10% FBS, penicillin (100 units/ml), streptomycin sulfate (100 mg/ml), and 1% NEAA in a humidified atmosphere of 5% CO_2_. Cells were stimulated with LPS (1 μg/ml) in the presence or absence of AS101 (0.5, 2 [μg/ml]), as indicated for specific experiments.

### Reagents

All media components were supplied by Biological Industries, Kibbutz Beit-Haemek, Israel; LPS (*E. coli*, 055:B5) (Sigma-Aldrich, Rehovot, Israel); AS101 was supplied by M. Albeck from the Department of Chemistry at Bar-Ilan University, in a solution of PBS, pH 7.4, and maintained at 4°C.

### Protein Isolation and Western Blotting

Cells were suspended with ice-cold lysis buffer containing 50 mM Tris(pH 7.5), 150 mM NaCl, 10% glycerol, 1% TritonX, 1 mM EDTA, 1 mM PMSF, 0.4 mM sodium vanadate, 5 mg/ml aprotinin, and 5 mg/ml leupeptin for 15 min on ice, and centrifuged at 14000 rpm for 10 min. Cell lysates were boiled for 5 min, electrophoresed on SDS-PAGE, and membranes were incubated with anti-iNOS, anti-IkB, anti-p65 (Santa-Cruz Biotechnology), anti-pIkB^ser32/36^(Cell Signaling) and actin (Sigma-Aldrich, Rehovot, Israel) antibodies. Blots were developed using horseradish peroxidase-conjugated secondary antibodies and the ECL detection system (Amersham-Pharmacia Biotech).

### Nuclear and cytosolic fractions preparation

Cells were suspended and homogenized with ice-cold lysis buffer containing: 10 mM Hepes (pH 7.4), 1.5 mM MgCl_2_, 10 mM KCl, 5 mg/ml aprotinin and 5 mg/ml leupeptin for 5 min. Suspended cells were centrifuged at 2400 rpm for 15 min, and the supernatants were centrifuged for 45 min at 14000 rpm. The cytosolic extracts were stored at -20°C. The nuclear pellet was resuspended and incubated for 45 min in lysis buffer containing: 20 mM Hepes (ph 7.4), 0.42 M NaCl, 1.5 mM MgCl_2_, 0.2 mM EDTA, 5 mg/ml aprotinin, and 5 mg/ml leupeptin. The nuclear lysate was centrifuged for 45 min at 14000 rpm and the fraction containing the soluble nuclear proteins was kept at -20°C.

### NO levels quantification

NO^-^_2 _was assayed by the Griess reaction, as a measure of NO production[19].

### IL-6 Quantification

IL-6 ELISA kit (R&D Systems, Minneapolis, MN) was used for the quantitative measurement of this cytokine in supernatants.

### ChIP

The ChIP assay was done using the Upstate-kit (Millipore, USA) according to manufacturer's instructions. Briefly, 1 × 10^6^/ml RAW 264.7 cells were treated with LPS (1 μg/ml) and AS101 (2 μg/ml) for 1 h. Formaldehyde (1%) was added to the culture medium, and after incubation for 10 min at 37°C, cells were lysed for 10 min at 4°C and were sonicated eight times for 15 s each. One third of the lysate was used as DNA input control. The remaining two-thirds were diluted 10-fold with Chip dilution buffer supplied within the commercial kit followed by incubation with an anti-p50 Ab or nonspecific control Ab (Santa-Cruz Biotechnology) overnight at 4°C. Immunoprecipitated complexes were collected using protein A-agarose beads. The precipitates were extensively washed and then incubated in the elution buffer (1% SDS and 0.1 M NaHCO_3_) at room temperature for 15 min. Cross-linking of protein-DNA complexes was reversed at 65°C for 4 h, followed by treatment with 10 mg/ml proteinase K for 1 h at 45°C. DNA was extracted with phenol/chloroform and precipitated with ethanol. Pellets were resuspended in TE buffer and subjected to PCR amplification using NFkB consensus site specific (forward:CAAGCCAGGGTATGTGGTTT; reverse:GCAGCAGCCATCAGGTATTT) and non-specific (forward: TTGGCACCATCTAACCTCAC, reverse:TGGTGTATCCTCATGCAAGG) primers (Hy-Labs, Israel) in iNOS promoter. The resulting product was separated by 1% agarose gel electrophoresis.

### Statistical Analysis

Results are expressed as the mean ± S.E. of triplicate experiments. Statistical significance of values was calculated using the Student's t-test. p < 0.05 was considered statistically significant.

## Results

### Effect of AS101 on LPS-induced iNOS expression, NO production and IL-6 secretion

**Figure 1:**
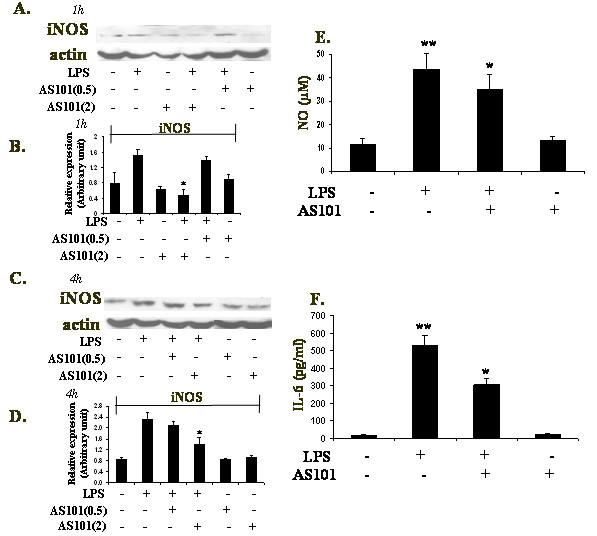
**Effect of AS101 on LPS-induced iNOS protein expression (A-D) and NO(E) and IL-6 (F) secretion**. (A) LPS-stimulated RAW264.7 cells (1 × 10^6^/ml) were treated with AS101(0.5 or 2 [μg/ml]) for 1 h (A) and 4 h (C). The iNOS level was analyzed by immunoblotting using anti-iNOS. Actin was used as an internal loading control. Bar graphs represent the quantitative densitometric value of the expressed protein vs actin: 1 h (B) and 4 h (D). *p < 0.05 vs LPS. Data shown are representative of three different experiments. (E-F) LPS-stimulated RAW264.7 cells (1 × 10^6^/ml) were incubated with AS101 (2 μg/ml) for 24 h. The culture supernatants were subsequently isolated and analyzed for nitrite and IL-6 levels. Data expressed as mean ± SE of four independent experiments. ** p < 0.05 vs. control, * p < 0.05 vs. LPS.

In order to induce an inflammatory response, similar to that observed in many pathophysiological conditions, LPS was used to stimulate the increase of iNOS and NO as well as IL-6 release from a macrophage cell line. In RAW264.7 macrophages, LPS (1 μg/ml) treatment resulted in the increase of iNOS protein expression starting from 1 h after initiation of treatment, whereas NO release was detectable after 24 h of LPS-stimulation (not shown). Co-treatment with AS101(2 μg/ml) markedly reduced iNOS induction at 1 h (Fig. 1A-B) and 4 h (Fig. 1C-D) after LPS stimulation vs LPS alone, while AS101(0.5 μg/ml)+LPS did not cause significant changes in iNOS expression vs LPS treated cells. To address whether inhibition of iNOS was paralleled by a reduction in NO release, NO production was determined in the form of nitrite in culture supernatants using the Griess reagent. Unstimulated cells produced low levels of NO_2_, while LPS stimulation considerably increased the amounts of nitrite secreted in culture supernatants (Fig. 1E). AS101 treatment of RAW264.7 cells significantly inhibited LPS-stimulated NO production (Fig. 1E), whereas AS101 alone did not cause significant changes in the NO levels. Moreover, LPS-induced IL-6 secretion was significantly down-regulated by AS101 treatment (Fig. 1F) while AS101 alone did not cause significant changes in the IL-6 levels. These results imply that AS101 may serve as anti-inflammatory agent through down-regulation in iNOS and NO as well as in IL-6 production. The protective anti-inflammatory capabilities of AS101 prompted us to examine the mechanism of action of this compound in our experimental system.

### AS101 down-regulates IKBα degradation and phosphorylation via different kinetics

**Figure 2:**
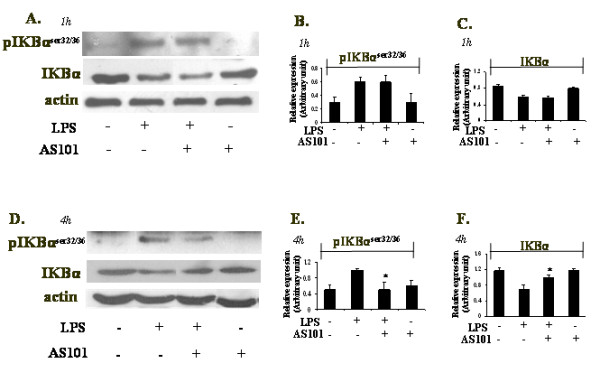
**Effect of AS101 on degradation and phosphorylation of IKBα in RAW264.7 macrophages**. (A,D) Cells were treated with LPS in the absence or in the presence of AS101 for 1 h (A) and 4 h (D). Total cellular proteins were prepared and immunoblotted using anti-pIKBα^ser32/36^, anti-IKBα and anti-actin. Bar graphs represent the quantitative densitometric value of the expressed protein vs actin: pIKBα^ser32/36 ^1 h (B) and 4 h (E), IKBα- 1 h (C) and 4 h (F). *p < 0.05 vs LPS. Data shown are representative of three different experiments.

Since IKB proteins degradation is an essential step for NFκB activation and expression of its target iNOS gene induced by LPS[2,20], AS101 effect on LPS-induced IKBα degradation was examined. IKBα degradation was detected with or without AS101 treatment 1 h after LPS stimulation (Fig. 2A, C), while IKBα phosphorylation (Fig. 2A, B) was not changed significantly in AS101 treated cells. Determination of IKBα degradation at 4 h after LPS stimulation showed significant inhibition of IKBα degradation in AS101 treated cells vs. those receiving LPS treatment alone (Fig. 2D, F). Furthermore, detection of IKBα phosphorylation revealed a clear inhibitory effect on LPS-induced IKBα phosphorylation in the presence of AS101 (Fig. 2D, E). This data suggest that the tellurium compound, AS101, down-regulates iNOS expression (Fig. 1) possibly through time dependent kinetics. While at 4 h after LPS stimulation, AS101 treatment prevented IKBα degradation and phosphorylation, at 1 h after LPS stimulation, inhibition of iNOS expression was observed in the presence of AS101 (Fig. 1), with no detection of inhibitory effect on IKBα degradation and phosphorylation.

### Effect of AS101 on LPS-induced NFkB translocalization and p50 DNA-binding

**Figure 3:**
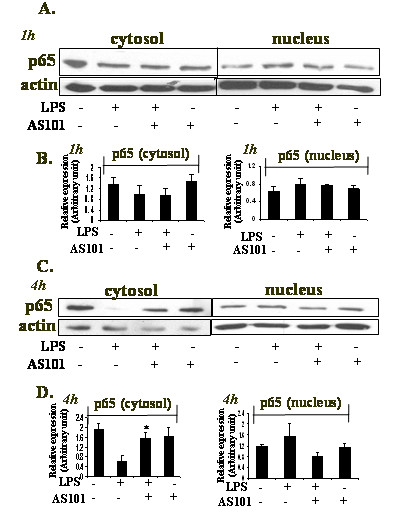
**Effect of AS101 on NFkB translocalization**. LPS-stimulated RAW264.7 cells (1 × 10^6^/ml) were treated with AS101 for 1 h (A) and 4 h (C). Cytosolic and nuclear extracts were immunoblotted using anti-p65 of NFκB and anti-actin. Extracts were immunoblotted using RCC1 indicating nuclear purity of the fractions (not shown). Bar graphs represent the quantitative densitometric value of the expressed protein vs actin: p65 1 h (B) and 4 h (D). *p < 0.05 vs LPS. Data shown are representative of three different experiments.

Since p65 is a major component in the NFkB complex activation, we examined p65 translocation to the nucleus by immunoblotting (Fig. 3). RAW264.7 cells were incubated with LPS in the presence or absence of AS101 for 1 h or 4 h. Translocation of p65 from the cytosol into the nucleus was evident after 1 h in the presence of LPS, whereas LPS-stimulated AS101-treated cells did not show significant changes vs LPS (Fig. 3A-B). At 4 h, LPS-activated cells demonstrated p65 translocation, while AS101 treatment abrogated this activity (Fig. 3C-D).

**Figure 4:**
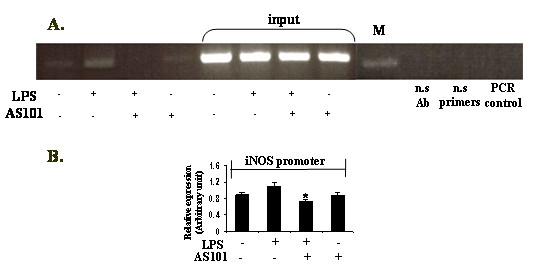
**Inhibition of p50 DNA-binding in iNOS promoter by AS101 treatment**. (A) ChIP analysis of LPS-stimulated RAW264.7 cells (1 × 10^6^/ml) treated with AS101 for 1 h. Bar graph represent the quantitative densitometric value of the p50 DNA-binding in the iNOS promoter vs input (B). *p < 0.05 vs LPS. Data are representative of three different experiments.

Since iNOS transcription requires NFkB activation through p50 DNA-binding to the indicated iNOS gene, chromatin immunoprecipitation assay was carried out using LPS-stimulated RAW264.7 extracts in the presence or absence of AS101. AS101 treatment attenuated p50 DNA-binding abilities to the iNOS promoter region in cells stimulated with LPS for 1 h (Fig. 4A-B). Collectively, these data imply that although AS101 did not inhibit NFkB nuclear translocation at 1 h, it prevented binding at the NFkB consensus site in the iNOS promotor following LPS induction.

## Discussion

In the present study, we attempted to elucidate the anti-inflammatory effects of the tellurium compound, AS101. We show that AS101 is an effective inhibitor of LPS-stimulated iNOS expression and NO secretion in RAW264.7 macrophages. The mechanism by which AS101 inhibits the expression of these inflammatory mediators appears to involve the NFkB pathway signaling. Interestingly, IkB phosphorylation and degradation and NFkB nuclear translocalization in LPS-stimulated macrophages were affected by AS101 treatment at different kinetics when tested at 1 h vs 4 h. Furthermore, AS101 treatment attenuated p50 subunit DNA-binding abilities in the iNOS promoter. Furthermore, the secretion of the inflammatory cytokine IL-6, regulated by the NFkB pathway, was significantly inhibited by AS101. These findings suggest that the tellurium compound, AS101, may prevent inflammation by suppressing NFkB mediated inflammatory genes.

The reactive free radical, NO, synthesized by iNOS is a major macrophage-derived inflammatory mediator, which is involved in various pathologies[21,22]. Moreover, it has been reported that IL-6 is a pro-inflammatory cytokine, regarded as endogenous mediator of LPS-induced fever[23]. AS101 treatment of LPS-activated RAW264.7 macrophages resulted in the decrease of IL-6 production as well as in the down-regulation of iNOS expression and NO secretion. NFκB is known to play a critical role in the regulation of cell survival genes and coordination of pro-inflammatory mediators such as iNOS and NO[2]. Therefore, the modulation of iNOS expression by AS101 prompted us to examine the effect of AS101 on this transcription complex activity. NFκB activation requires IKBα phosphorylation, which then targets IKBα for ubiquitination and degradation [20]. Interestingly, IKBα phosphorylation and degradation followed by NFkB nuclear translocalization was affected differently by AS101 treatment, depending on the time point studied. Thus, although AS101(2 μg/ml) inhibits iNOS expression at both 1 h and 4 h (Fig. 1A, C), it does not affect neither IKBα phosphorylation nor its degradation at 1 h (Fig. 2A-C). Furthermore, NFkB nuclear translocation was neither affected by AS101 at that time point (Fig. 3A-B). Nevertheless, at 4 h, all these processes were affected by AS101: the compound both prevented IKBα phosphorylation and degradation (Fig. 2D-F) and inhibited NFkB nuclear translocation (Fig. 3C-D).

The molecular pathways involved in the regulation of iNOS expression occur largely at a transcriptional level and appear to be immensely heterogeneous, with particular mechanisms invoked in specific cell types[24]. However, a common signaling molecule involved in these diverse pathways is the ubiquitous inflammatory transcription factor, nuclear factor NFkB [24]. Based on this evidence, and given the inhibitory effect of AS101 on NFkB activation at 4 h, the lack of modulation of the NFkB pathway by AS101 at 1 h, concomitantly with the inhibition of iNOS at that time point, prompted us to further explore the mechanism of iNOS inhibition at 1 h. The results of the Chip assay revealed that treatment with AS101 of LPS-stimulated RAW264.7 macrophages attenuated p50-binding to the iNOS promoter region vs. LPS treatment alone (Fig. 4).

Accumulated evidence suggests that much of the biological activity of organotellurium compounds is directly related to their specific chemical interactions with cysteine thiol residues. The Te(IV)-thiol chemical bond may lead to conformational change or disulfide bond formation in a specific protein, possibly resulting in the loss of its biological activity, if the thiol residue is essential for that function. Indeed, we demonstrated that AS101 and other TeIV-compounds specifically inactivate cysteine proteases [14-16], while exhibiting no effect on the other families of serine-, aspartic and metalloproteases, in good agreement with the predictions of their unique Te(IV)-thiol chemistry. Furthermore, the proteolytic activity of the inactivated cysteine proteases could be restored by reducing agents further supporting the suggestion that the inactivation process involves oxidation of the catalytic thiol to a disulfide[14]. Furthermore, neuroprotection exerted by AS101 in both Parkinson's disease models[16] and ischemic stroke[25] were shown to be mediated by the Te(IV) redox chemistry of the compound. Likewise, the protective mechanism of AS101 against homocysteine toxicity was shown to be directly mediated by its chemical reactivity, whereby AS101 reacted with homocysteine to form homocystine, the less toxic disulfide form of homocysteine[25]. These marked redox potential of AS101 may account for the aforementioned anti-inflammatory effects of the compound.

The critical step in NF-kB activation is IkBα phosphorylation at Ser32 and Ser36 by IkB kinase complex [26] while both IKKs contain a cysteine at 179 in their activation loop. Based on the evidence that NFκB has a well-conserved cysteine residue in its p50-subunit together with location of NFκB recognition consensus-binding site in the iNOS promoter - modulation of NFκB activity may be carried out by redox regulation in a great part through a decrease in DNA-binding activity due to redox-sensitive cysteine residues[27,28].

Thus, the effect of AS101 in our experimental system may be mediated by inhibition of two steps in the NFκB pathway by modifying specific cysteine residues in IKKα and in the p50-subunit resulting in the inhibition of nuclear translocation and DNA-binding to the iNOS promoter. Nevertheless these effects are exerted at different kinetics. I. At 1 h, AS101 probably enters the nucleus and may interfere with the DNA-binding ability of the NFκB complex resulting in the inhibition of iNOS expression. Because tellurium readily forms complexes such as Rs-Te-SR or Rs-Te with reactive sulfhydryl groups in proteins, such derivatives could account for the observed inhibition of p50-binding to its DNA targets by the reactive tellurium compound. II. At 4 h inhibition of iNOS expression by AS101 may be attributed to the compound's activity in the cytosol inhibiting IKKα phosphorylation, degradation and NFκB nuclear translocation.

The NFκB complex functions as a key factor in inflammation. AS101 treatment inhibits NFκB activities and thereby acts as an anti-inflammatory agent in NFκB target genes such as iNOS and NO formation as well as IL-6 production. Moreover, AS101 has been shown to have therapeutic effects in various experimental animal models without obvious side effects and has shown excellent safety profile in human clinical trials. The investigation of therapeutic activities of tellurium compounds is scarce in the literature, despite the relative abundance of tellurium in the human body. Over the last decade, there has been an increased appreciation for the role of redox chemistry in the regulation of biological systems. Understanding the mechanism of thiol modifying tellurium compounds such as AS101, currently used in phase II/III clinical trials, that blocks multiple steps in the NFκB signaling pathway, may lead to the development of more effective therapies for inflammatory diseases.

## Abbreviations

(NO): Nitric Oxide; (iNOS): inducible Nitric Oxide Synthase; (NFκB): Nuclear transcription factor kappa-B; (AS101): Ammonium trichloro(dioxoethylene-O,O') tellurate; (LPS): Lipopolysaccharide; (ChIP): Chromatin immunoprecipitation.

## Competing interests

The author(s) declare that, except for income received from their primary employer, no financial support or compensation has been received from any individual or corporate entity over the past three years for research or professional service and there are no personal financial holdings that could be perceived as constituting a potential conflict of interest.

## Authors' contributions

MB conceived of the study, performed the experiments and carried out the majority of the assays, performed the statistical analysis, participated in the design and coordination of the study, and drafted the manuscript.

GH carried out the IL-6 ELISA, participated in the statistical analysis and in the coordination of the study.

MA participated in the design of the study, its coordination and drafted the manuscript.

BS conceived of the study, participated in the design and coordination of the study, and drafted the manuscript.

All authors read and approved the final manuscript.
